# Trisomy 13 and Massive Fetomaternal Hemorrhage

**DOI:** 10.14740/jocmr2169w

**Published:** 2015-05-08

**Authors:** Ryoko Matsui, Shunji Suzuki, Marie Ito, Yusuke Terada, Sakae Kumasaka

**Affiliations:** a Department of Obstetrics and Gynecology, Japanese Red Cross Katsushika Maternity Hospital, Tokyo, Japan

**Keywords:** Massive fetomaternal hemorrhage, Trisomy 13, Intervillous thrombosis, Preeclampsia

## Abstract

This is the first case report of trisomy 13 complicated by massive fetomaternal hemorrhage (FMH). A pale male infant weighing 2,950 g was delivered with low Apgar scores by emergency cesarean section due to non-reassuring fetal status. The umbilical arterial pH and hemoglobin level were 6.815 and 6.9 g/dL (normal: 13 - 22 g/dL), respectively. The maternal hemoglobin-F and serum alpha-fetoprotein levels were 6.0% (normal: < 1.0%) and 1,150 ng/mL (4.1 multiple of median), respectively. The neonate was diagnosed as having trisomy 13 by a subsequent chromosome examination. In the placenta, massive intervillous thrombosis was observed microscopically. This placental finding has been reported to be associated with both preeclampsia and massive FMH. In addition, the incidence of preeclampsia in pregnancies complicated by trisomy 13 has been reported to be significantly higher than normal karyotype populations. Therefore, the current finding may support the association between trisomy 13 and the incidence of massive FMH.

## Introduction

Fetomaternal transplacental hemorrhage of 30 mL or more has been reported to be in just 3 of 1,000 pregnancies [1]. Massive fetomaternal hemorrhage (FMH) has been defined as bleeding of greater than 50 - 150 mL of fetal blood in the maternal circulation, which can cause fetal anemia or perinatal death [1-4]. For example, the incidences of hemorrhage of 80 mL or more and 150 mL or more have been observed to be 1 in 1,150 and 1 in 2,810 pregnancies, respectively [4]. Some possible risk factors associated with massive FMH have been reported as follows: 1) external factors such as abdominal trauma, external version and amniocentesis, and 2) placenta tumor such as choriocarcinoma [1-4]. We present here a case of massive FMH in the fetus with trisomy 13 without these risk factors.

## Case Report

A 38-year-old woman, gravida 2, para 1, visited the hospital because of a marked decrease in fetal movement that began 2 days ago at 38 weeks and 1 days’ gestation. Fetal heart rate (FHR) monitoring showed decreased long-term variability of the FHR (base line = 130 bpm) without acceleration. At this time, her blood pressure and body temperature were 140/90 mm Hg and 36.3 °C, respectively. The patient was immediately admitted and a cesarean section was performed. A pale male infant weighing 2,950 g was delivered, with Apgar scores of 2 and 3 at 1 and 5 min, respectively. The arterial pH, hemoglobin level and reticulocytes of the umbilical cord were 6.815, 6.9 g/dL (normal: 13 - 22 g/dL) and 11.7% (normal: 3-7%), respectively. The neonate was recovered by hemo-transfusion of packed red blood cells. At this time, the maternal hemoglobin-F and serum alpha-fetoprotein levels were 6.0% (normal: < 1.0%) and 1,150 ng/mL (4.1 multiple of median), respectively. The neonate was complicated by corpus callosum agenesis, cleft lip and palate, polydactyly and polycystic kidneys and was diagnosed as having trisomy 13 by a subsequent chromosome examination. Thirty days after the delivery, the maternal hemoglobin-F and alpha-fetoprotein levels decreased to normal. Based on these findings, she was diagnosed to be a case of trisomy 13 complicated by massive FMH. The estimated FMH volume was 140 mL (46 mL/kg). The placenta weighed 450 g. Microscopically, massive intervillous thrombosis in the placenta was confirmed.

## Discussion

To our knowledge, this is the first case report of trisomy 13 complicated by massive FMH. Massive FMH and trisomy 13 are both rare and severe complications in fetuses. In this case, these two severe complications might have concurrent accidentally. However, we are able to perform further consideration about the mechanisms associated with the placenta of trisomy 13 leading to the incidence of FMT.

The incidence of preeclampsia in pregnancies complicated by trisomy 13 has been reported to be significantly higher than those in trisomy 18 and normal karyotype populations [5]. The placental findings that showed avascular edematous cystic villi have been suggested to be associated with angiogenic imbalance involved in the pathogenesis of preeclampsia in trisomy 13 pregnancies [6]. The intervillous thrombosis in the placenta and the thrombosis in the fetal circulation were found to have significantly higher rates in the preeclamptic pregnancies [7]. The placenta complicated by massive FMH has been also reported to show an increased frequency of intervillous thrombosis like the current case [8]. In the current case, the mother did complicated by typical preeclampsia; however, her blood pressure on admission was 140/90 mm Hg. The current finding may support the association between trisomy 13 and the incidence of massive FMH [5-8].

To date, if trisomy 13 had been turned intrauterine fetal death, there may be the possibilities that the reason for the fetal demise has not been properly validated. However, the elucidation of the mechanisms is needed by accumulation of the same case reports.
